# On the Evolution of Hexose Transporters in Kinetoplastid Potozoans

**DOI:** 10.1371/journal.pone.0036303

**Published:** 2012-05-02

**Authors:** Claudio Alejandro Pereira, Ariel Mariano Silber

**Affiliations:** 1 Instituto de Investigaciones Médicas “Alfredo Lanari," Ciudad Autónoma de Buenos Aires, Argentina; 2 Departamento de Parasitologia, Instituto de Ciências Biomédicas, Universidade de São Paulo, São Paulo, São Paulo, Brasil; State University of Campinas, Brazil

## Abstract

Glucose, an almost universally used energy and carbon source, is processed through several well-known metabolic pathways, primarily glycolysis. Glucose uptake is considered to be the first step in glycolysis. In kinetoplastids, a protozoan group that includes relevant human pathogens, the importance of glucose uptake in different phases of the life cycles is well established, and hexose transporters have been proposed as targets for therapeutic drugs. However, little is known about the evolutionary history of these hexose transporters. Hexose transporters contain an intracellular N- and C- termini, and 12 transmembrane spans connected by alternate intracellular and extracellular loops. In the present work we tested the hypothesis that the evolutionary rate of the transmembrane span is different from that of the whole sequence and that it is possible to define evolutionary units inside the sequence. The phylogeny of whole molecules was compared to that of their transmembrane spans and the loops connecting the transmembrane spans. We show that the evolutionary units in these proteins primarily consist of clustered rather than individual transmembrane spans. These analyses demonstrate that there are evolutionary constraints on the organization of these proteins; more specifically, the order of the transmembrane spans along the protein is highly conserved. Finally, we defined a signature sequence for the identification of kinetoplastid hexose transporters.

## Introduction

The order Kinetoplastidae consists of flagellated protozoans that have a peculiar mitochondrial DNA-containing structure called kinetoplast. This group includes species of parasitic protozoa of the genera *Trypanosoma* and *Leishmania*, which cause severe human disease. Two species of the genus *Trypanosoma*, *T. brucei* and *T. cruzi*, are the causative agents of sleeping sickness and Chagas' disease, respectively. Species of the *Leishmania* subgenera *Leishmania* and *Viannia* are the causative agents of a group of diseases known together as leishmaniasis. Together, these parasites affect approximately 25 million people in endemic areas all over the world, with an estimated population of more than 350 million people at risk of acquiring these infections. Unfortunately, the drugs used to treat human infections by these parasites are unsatisfactory: their low therapeutic efficiency, high toxicity, and the appearance of resistant strains constitute serious drawbacks that remain to be overcome [1].

Kinetoplastid protozoa usually have a complex life cycle, alternating between one or more hosts and frequently moving between several territories with different nutrient compositions inside their hosts (i.e., different regions of the insect midgut). The ability of these cells to deal with these environmental changes is reflected by their flexible metabolism, which allows them to use glucose or amino acids (mainly proline) as their main carbon and energy source [2], [3]. The cells usually consume amino acids only when they are subjected to glucose deprivation conditions [4], [5], supporting the idea that glucose consumption is the default option and emphasizing the relevance of this carbohydrate for these organisms [6].

Transporters are essential proteins in most cells and are among the first molecules involved in the detection of their substrates in the extracellular medium [7]. In trypanosomatids, glucose uptake can be considered to be the first regulated step of glycolysis [8]. These findings led to the early study of glucose uptake pathways in pathogenic trypanosomatids and to molecular studies of hexose transporters in *T. brucei*, *T. cruzi* and *Leishmania* spp. parasites [9]. The relevance of glucose metabolism has been particularly well demonstrated in these trypanosomatids, and hexose transporters and several stages of glycolysis have been proposed as drug targets [10], [11]. More recently, with the advance of the *T. brucei*, *T. cruzi* and *L. major* (TriTryp) genome projects, additional hexose transporter genes belonging to other pathogenic and non-pathogenic kinetoplastids have been annotated [12], and some of them have been experimentally validated [8]. All of the glucose transporters described to date in kinetoplastids are related to solute carrier family 2, also known as facilitated glucose transporter member 1 (SLC2A1) or GLUT1, belonging to the Major Facilitator Superfamily (2.A.1, MFS). Although we have a considerable amount of information about the GLUT1 family, little is known about the evolution of these peculiar proteins [13].

GLUT1 is a glucose transporter family that is broadly distributed across Eukarya and Bacteria [14]. The structure of these facilitative hexose permeases is supported exclusively by twelve transmembrane spans, which are connected by extracellular and intracellular loops [15], [16]. Because the transport activity of these proteins relies on their transmembrane regions, we hypothesize here that the connecting loops have fewer constraints with regard to amino acid substitution during evolution. Following this rationale, we hypothesize that the transmembrane regions of hexose transporters constitute evolutionary modules with more constrained variation patterns than the extracellular and intracellular loops. In the present work we used the kinetoplastid hexose transporter sequences available in the TriTryp database [17] to analyze the evolutionary history of these proteins in species belonging to the genera *Trypanosoma* and *Leishmania*, with particular focus on the transmembrane regions. The results presented herein show for the first time that clusters of consecutive transmembrane spans constitute evolutionary modules.

## Materials and Methods

### Sequence retrieval and domain identification

All available amino acid sequences annotated as hexose/glucose transporters were recovered from the TriTryp database version 3.2 (http://tritrypdb.org/tritrypdb) [17]. The species and accession numbers of the sequences used are listed in Table 1. Only sequences corresponding to a single allelic copy per species were chosen to be included in the present analysis. The initial search for constructing the starting-up database was performed as follow. Due to the presence of two haplotypes (Esmeraldo and non-Esmeraldo) in the available genome data from the *T. cruzi* CL Brener strain, only one representative gene was analyzed; *T. cruzi* Esmeraldo if it exists, otherwise *T. cruzi* non-Esmeraldo if it exists, otherwise *T. cruzi* unassigned genes. In the case of *T. brucei*, non-redundant genes were also analyzed considering the representative gene this corresponding to *T. brucei* strain TREU927 if it exists, otherwise *T.brucei* strain 427 if it exists, otherwise *T. brucei gambiense*. Sequences from other *Trypanosoma spp*. and *Leishmania spp*. were included if their identity were less than 98%. The obtained sequences were curated initially on the basis of their similarities to glucose transporters by using the online version of BLASTp at the NCBI (http://www.ncbi.nlm.nih.gov/BLAST/). BLASTp was run under default parameters using the non-redundant protein sequence database. The sequences were further analyzed for the position of the 12 transmembrane spans with SOSUI v1.11 [18], TMHMM v2.0 [19], TMPRED v1.0 (http://www.ch.embnet.org/software/TMPRED_form.html) and HMMTOP v2.0 (http://www.enzim.hu/hmmtop/). The predicted transmembrane regions were recovered as independent sequences for further analysis. Assemblies and analysis of the amino acid sequence data were carried out using the software package Vector NTI v. 10.3.0 (Invitrogen, California – USA).

**Table 1:** Description of the main characteristics of the used sequences.

AN	Species	Length	TMS	NTP/CTP	NTL	CTL
LbrM.33.0290	*L. braziliensis*	617	12	I/I	94	27
LbrM.35.6490	*L. braziliensis*	531	12	I/I	20	24
LinJ.36.6550	*L. infantum*	567	12	I/I	46	32
LinJ.36.6560	*L. infantum*	653	12	I/I	135	32
LmjF.33.0290	*L. major*	594	12	I/I	73	24
LmjF.36.6280	*L. major*	568	12	I/I	46	32
LmjF.36.6290	*L. major*	561	12	I/I	45	26
LmjF.36.6300	*L. major*	653	12	I/I	135	32
LmxM.32.0290	*L. mexicana*	627	12	I/I	107	24
LmxM.36.6280	*L. mexicana*	566	12	I/I	48	32
LmxM.36.6290	*L. mexicana*	567	12	I/I	49	32
LmxM.36.6300	*L. mexicana*	610	12	I/I	88	32
Tb427.04.2290	*T. brucei*	552	12	I/I	51	07
Tb427.10.8450	*T. brucei*	527	12	I/I	43	30
Tb427.10.8530	*T. brucei*	529	12	I/I	41	31
Tc00.1047053506355.10	*T. cruzi*	544	12	I/I	34	47
TcIL3000.10.7320	*T. congolense*	525	12	I/I	40	29
TvY486_0402140	*T. vivax*	558	12	I/I	48	09
**Ecoli_P02920 (Lac Y)**	***E. coli***	** 417**	**12**	**I/I**	**07**	**19**

**AN:** Accession Numbers; **TMS:** number of transmembrane spans in each sequence; **NTP:** N-terminal position; **CTP:** C- terminal position; **NTL:** N- terminal length; **I:** intracellular; **E:** extracellular.

### Phylogenetic and other bioinformatic analyses

Phylogenetic analyses were performed using Molecular Evolutionary Genetics Analysis (MEGA) v5.05 [20], [21], [22]. Briefly, the evolutionary history was inferred with the maximum likelihood method with a JTT matrix-based model. The bootstrap consensus tree inferred from 500 replicates was taken to represent the evolutionary history of the sequences analyzed. Branches corresponding to partitions reproduced in fewer than 50% of bootstrap replicates were collapsed. Initial tree(s) for the heuristic search were obtained automatically as follows. When the number of common sites was lower than 100 or less than one-fourth of the total number of sites, the maximum parsimony method was used; otherwise, the BIONJ method with the MCL distance matrix was used. The trees were drawn to scale, with branch lengths measured in the number of substitutions per site. All positions containing gaps and missing data were eliminated. The average evolutionary divergence over all sequence pairs was also calculated using the JTT matrix-based model involving all amino acid sequences in each case. Similarity analyses based on the consensus sequence were conducted using the ClustalW algorithm [23]. For the identification of possible specific signatures, all sequences were scanned using Multiple Em for Motif Elicitation (MEME) v4.6.1 [24].

**Figure 1:**
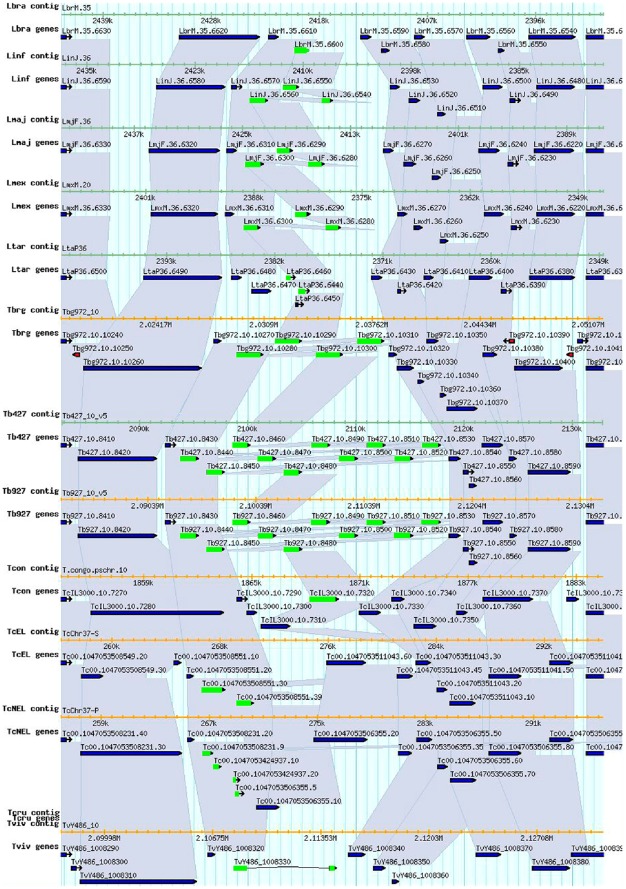
Hexose transporters synteny analysis. The genomic contexts of the *Trypanosoma* spp and *Leishmania* spp hexose transporters were analyzed. The figure represent one syntenic orthologous group (highlighted in green) comprising the genes LinJ.36.6550 (and its paralog LinJ.36.6560), LmjF.36.6280 (and its paralogs LmjF.36.6290-LmjF.36.6300, called lmgt1-3), LmxM.36.6280 (and its paralogs LmxM.36.6290-LmxM.36.6300), TcIL3000.10.7320, and Tb427.10.8450 (and its paralog Tb427.10.8530, called THT1). This group of genes has expanded into 10 tandem duplications. Lila shadows indicates the syntenic genes and all the sequences are represented by their systematic ID. The synteny analyses and scheme was made at the TriTrypDB (http://tritrypdb.org/tritrypdb).

## Results

Our search for all amino acid sequences annotated as hexose or glucose transporters in the TriTryp database returned 57 sequences (Table 1). To eliminate redundancies all allelic copies (>98% identity), truncated sequences and pseudogenes were discarded. Of the remaining 18 sequences distributed among 7 species of the genera *Trypanosoma* and *Leishmania*, the functions of 10 had been experimentally verified, while the functions of the other 8 had been inferred from sequence data [9], [25], [26], [27], [28], [29], [30], [31].

The analyzed genes fall into three syntenic orthologous groups. The first group comprises the genes LinJ.36.6550 (and its paralog LinJ.36.6560), LmjF.36.6280 (and its paralogs LmjF.36.6290-LmjF.36.6300, called lmgt1-3), LmxM.36.6280 (and its paralogs LmxM.36.6290-LmxM.36.6300), TcIL3000.10.7320 and Tb427.10.8450 (and its paralog Tb427.10.8530, called THT1). This group of genes has expanded into 10 tandem duplications (Figure 1). The second group comprises the orthologs LbrM.33.0290, LmjF.33.0290 and LmxM.32.0290, and the third group comprises Tb427.04.2290 and TvY486_0402140. LbrM.35.6490 and Tc00.1047053506355.10 have no orthologs.

All of these sequences were analyzed for the presence of putative transmembrane regions. To make the analysis more robust, four different algorithms were used to define the most probable transmembrane regions and the orientation of the N- terminal region of each sequence. As previously mentioned, all of the hexose transporter sequences analyzed in the present study belong to the major facilitator superfamily (MFS) [32].

Because the lactose permease (LacY) from *Escherichia coli* is a well-known member of this superfamily and its structure has been extensively studied [33], [34], we used it as a model for inferring the structural aspects of trypanosomatid transporters. In all cases, 12 transmembrane domains could be detected (Table 1). These domains were then excised from the surrounding sequence for further analysis. In this way, we obtained 216 fragments, which were subsequently treated as separate sequences (Dataset S1).

**Figure 2:**
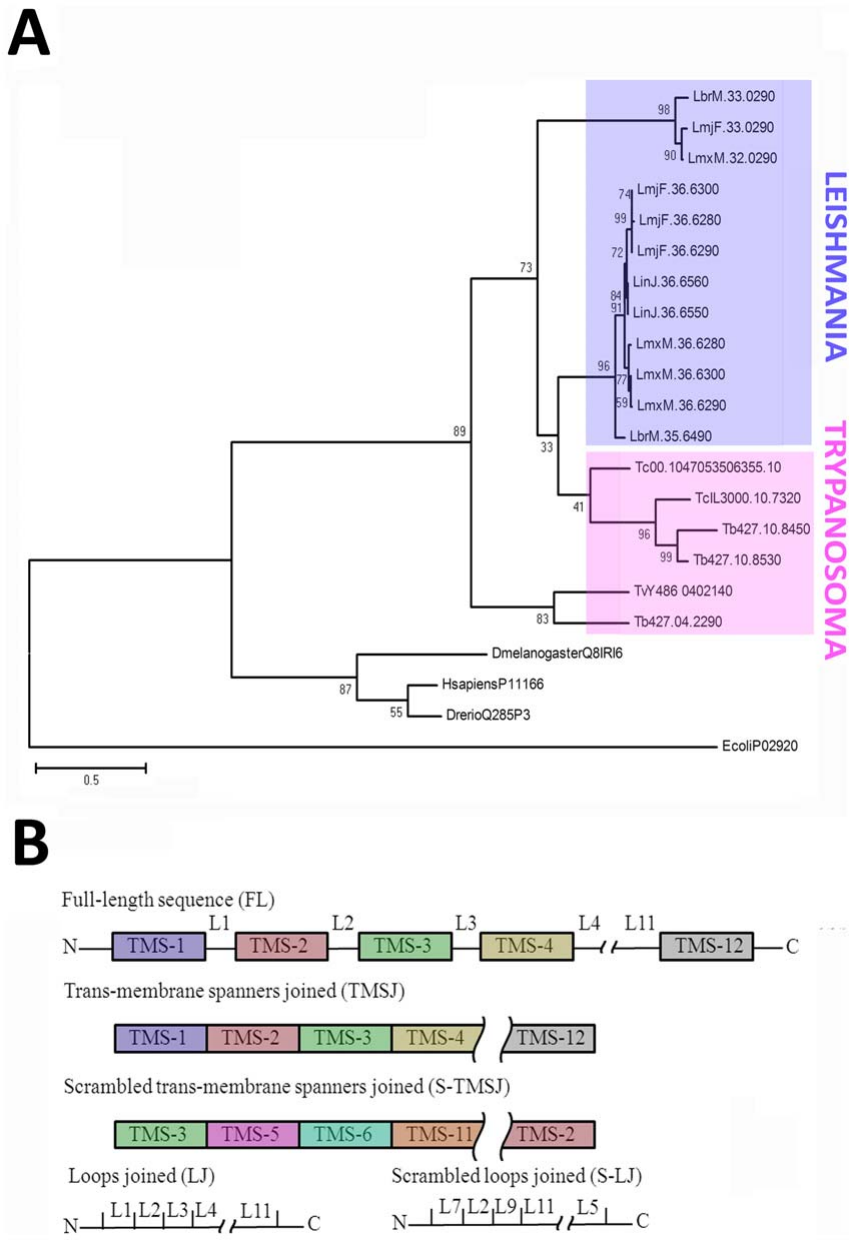
Phylogeny of the hexosetransporters. **A:** Evolutionary tree constructed using the full-length amino acid sequences of the kinetoplastid hexose transporters. Sequences coding for glucose transporters of *Homo sapiens*, *Drosophila melanogaster, Danio rerio* and *Escherichia coli* were used as outgroups to root the tree. A maximum likelihood method based on the JTT matrix-based model was used, with 500 bootstrap replicates. The tree with the highest log likelihood (−7654.7770) is shown. The tree is drawn to scale, with branch lengths measured in the number of substitutions per site. The kinetoplastid hexose transporters segregate into two main groups, which correspond to the genera *Trypanosoma* and *Leishmania*. **B:** Schematic representation of the structure of the sequences used. **FL:** full-length sequence, **TMS:** transmembrane span, **L:** loops connecting the TMSs, **TMSJ:** sequence containing contiguously ordered TMSs, **LJ:** sequence containing contiguously ordered Ls, **S-TMSJ:** scrambled TMSJ, **S-LJ:** scrambled SJ.

To infer the evolutionary history of trypanosomatids hexose transporters, we submitted their amino acid sequences to phylogenetic analysis by the maximum likelihood method using 500 bootstrap replicates (Figure 2A). To root the trees, sequences corresponding to the hexose transporter family GLUT1 from several unrelated organisms, that is, *Homo sapiens*, *Drosophila melanogaster*, *Danio rerio* and *Escherichia coli* Lac Y, were used. Among the trypanosomatid sequences, we observed that two sequences, Tb427.04.2290 and TvY486_0402140, branched earlier than the rest. The remaining 16 sequences were mainly distributed into two branches: one branch grouped the only *T. vivax* sequence with one out of three *T. brucei* sequences, while the other branch contained all other transporters. This second branch was divided into two main clusters, which corresponded to the genera *Leishmania* and **Trypanosoma.**

**Figure 3:**
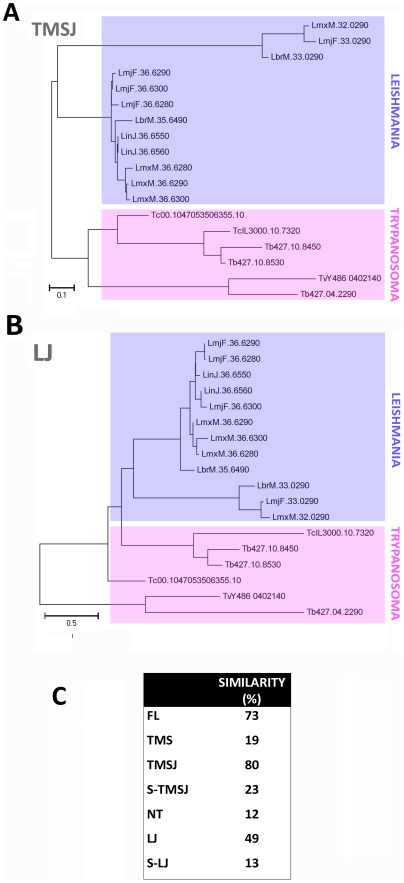
Evolutionary trees of transmembrane spans and loops. Evolutionary tree using the TMSJ (**A**) or LJ (**B**) sequences. A maximum likelihood method based on the JTT matrix-based model was used, with 500 bootstrap replicates. Trees with the highest log likelihood (−3067.0851 and −4129.5431, respectively) are shown. Trees are drawn to scale, with branch lengths measured in the number of substitutions per site. In both cases, sequences segregate into two main groups corresponding to the genera *Trypanosoma* and *Leishmania*. **C:** The mean distances for each group of sequences analyzed (FL, TMS, TMSJ, S-TMSJ, NT, LJ and S-LJ) were calculated and are presented as similarity values.

Hexose transporters, similar to other integral membrane proteins, are divided into defined modules, i.e., the transmembrane regions, which are essential for protein activity. Consequently, we hypothesized that the evolution of the transmembrane domains should be more constrained than that of the whole molecule. To test this hypothesis, for each full-length protein, we generated the following: 1) a sequence in which we removed the intracellular and extracellular loops connecting the transmembrane regions (loops or L), and 2) a sequence in which the transmembrane spans (TMS) were removed. Thus, for each full-length original sequence, we obtained one sequence containing only the transmembrane spans (transmembrane spans joined, or TMSJ) and one sequence containing only the loops connecting the transmembrane regions (loops joined, or LJ) (Figure 2B). These sequences were then resubmitted to phylogenetic analysis by the maximum likelihood method, again using 500 bootstrap replicates (Figures 3A and B). The resulting trees were similar to those obtained when the full-length protein sequences were used, showing two defined clusters that separated the sequences by genus. This finding suggests that, in qualitative terms, the evolution of the transmembrane regions did not differ from that of the full-length sequence. We also analyzed the mean similarity among the full-length, TMSJ and LJ sequences. Percent similarity values of 73% and 80% were calculated for the full-length and TMSJ sequences, respectively, while the values obtained for the variable N- termini and the LJ sequences were 12% and 49%, respectively (Figure 3C). Taken together, these data show that the transmembrane regions of these sequences are more stable over evolutionary time than the whole molecule, the N- terminus or the LJ. Thus, these results support the idea that the transmembrane domains of hexose transporters are the most evolutionarily constrained modules in these molecules.

**Figure 4:**
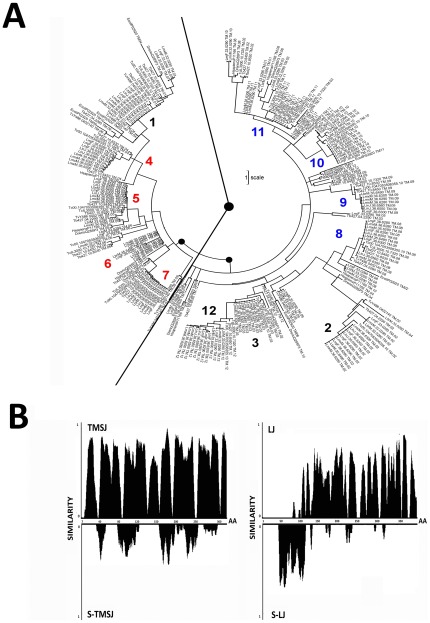
Evolution of each transmembrane spanindividually. **A:** Evolutionary tree using the pool of individual TMS sequences. A maximum likelihood method based on the JTT matrix-based model was used, with 500 bootstrap replicates. The tree is drawn to scale, with branch lengths measured in the number of substitutions per site. The tree with the highest log likelihood (−3119.4504) is shown. Two major superclusters are separated by a solid line in which numbers indicate the main positions for the grouped TMSs. Colored numbers indicate consecutive TMSs from each supercluster. Scale bar corresponds to distance expressed in amino acid substitutions per site. **B:** Similarity as a function of position was plotted to compare TMSJ against S-TMSJ, and LJ against S-LJ.

From the data obtained, two models can be proposed for the evolution of hexose transporters in trypanosomatids: 1. The proteins evolved as a whole from a full-length ancestor, or 2. The proteins evolved through the independent evolution of the constituent modules. To distinguish between these models, the transmembrane span regions (TMS) or the N- and C- termini together with the loops connecting the transmembrane regions (loops, or L) of all sequences were extracted and numbered consecutively (based on the position they occupy in the original sequence). Thus, the sequences designated as “1" were located closest to the N- termini (Figure 2B). For this work, all 216 transmembrane regions and 234 loops were treated individually as single evolutionary units and were analyzed by the maximum likelihood method. TMSs from the sequences used as outgroups, including LacY, were also analyzed. Interestingly, we observed that most of the TMS sequences segregated into two major groups, which branched close to the base of the tree (see Figure 4A). The consecutively numbered spans formed two coherent superclusters organized as follows: A) 1, 4–7, and B) 2–3, 8–11 and 12 (Figure 4A).

The same method was used to analyze the loops connecting the transmembrane regions. Unlike what occurred with the TMS sequences, we observed that the loop sequences segregated into randomly distributed clusters (Figure S1). Due to the particularly high levels of divergence in these sequences, the use of distance estimation analysis by overall mean distances (based on the JTT algorithm) failed in detecting common sites among the sequences. Taken together, these results support the idea that these molecules have modules of evolution, consisting of single transmembrane regions or discrete groups of transmembrane regions.

We next sought to examine the evolution of the order in which these modules are arranged. To approach this problem, we first made identity plots for sequences in which the transmembrane spans were joined in sequential order (TMSJ) or randomly scrambled (S-TMSJ, see Figure 2B). We observed high levels of identity inside each transmembrane span and low levels in the interface regions (the position at which one span ends and the next one begins). However, when the order of the transmembrane regions was scrambled, we observed that identities as a function of amino acid position were completely lost: the mean global identity values diminished from 71.9% for TMSJ to 20.4% for S-TMSJ. When the same analysis was performed on the connecting loops, the identity values decreased for both the LJ and S-LJ sequences (36.5 and 12.2%, respectively) (Figure 4B), indicating a small contribution of the order of the L regions to global identity (see values in Figure 3C). This result strongly indicates that, in addition to the fact that individual or clusters of transmembrane regions act as evolutionary modules, their order inside the molecule is critical for functional hexose transporters. To estimate the evolutionary divergence between TMSs in a single sequence and between TMSs at the same location in different sequences, the TMSs were grouped by constructing a matrix **M**[i,j], where i represents a sequential array of TMSs from the same molecule (TMS-1, …, TMS-12) and j represents TMS-i for each of the 18 sequences under analysis (TMS-1,1, …, TMS-1,18) (Table S1). The whole matrix divergence was calculated to be 7.7 arbitrary units. This value was similar to those obtained within each row and among rows and to that calculated among columns. Interestingly, the mean divergence within each column was 1.45; the column corresponding to TMS-1 was the only one with a high divergence value (8.46). Interestingly, the N- and C- termini were the most divergent regions along the whole molecule. These data indicate that the TMSs corresponding to the same positions in different proteins are the most conserved evolutionary units of kinetoplastid hexose transporters. In addition, although it is likely that these proteins evolved by internal TMS duplications, no traces of this process remain detectable in the sequences under analysis. This conclusion is supported by our analysis of internal amino acid repeats in these proteins using four different algorithms, none of which found evidence of TMS duplications.

The presence of conserved regions in the hexose transporters studied could prove valuable for identifying critical regions related to the protein activity. All of the sequences were analyzed using the Multiple Em for Motif Elicitation (MEME) algorithm (http://meme.nbcr.net/). This analysis identified the motif QLTGINA (which we refer to as the “GINA" motif), which was primarily present in TMS-6 and TMS-7 (Figure S2).

## Discussion

The kinetoplastid hexose transporters have low but significant identity to members of the mammalian GLUT1 family [35]. For those hexose transporters that have been cloned and have had their activity demonstrated, it is predicted that the proteins contain 12 transmembrane spans [25], [26], [27], [31], [36], [37], [38], [39], [40], [41]. However, this should not be taken as a rigid rule. As the genome projects for more trypanosomatids progress, new sequences are being annotated as putative hexose transporters, some of which have 10 or 11 transmembrane regions predicted by validated bioinformatic tools [12]. In the present study, we found 57 hexose transporters annotated for seven pathogenic and non-pathogenic kinetoplastid species (listed in Table 1). After curating the obtained sequences, we arrived at a total of 18 sequences, all them bearing 12 transmembrane spans. Because glucose transporters are essential for most living cells, new ones will likely be found in other trypanosomatid genomes. In this study, we detected a signature for kinetoplastid hexose transporters, the “GINA" motif (QLTGINA), which could be useful for detecting putative hexose transporters in newly sequenced kinetoplastids.

The probable structure of the kinetoplastids hexose transporters was classically based on that of the GLUT1 members of the GLUT family [9], which belong to the major facilitator superfamily (MFS) [13]. Most of members of this superfamily are predicted to contain 12 transmembrane helices based on the hydropathy plot for GLUT1 [13] which was first described by Mueckler et al. [42]. One of the best studied members of this group is the *E. coli* LacY protein, the structure of which was resolved in detail by crystallographic studies [33], [34]. This protein consists of 12 transmembrane regions, which act as the structural support for the protein's transport activity. In the LacY crystallography model, it was established that the spans are not arranged in space in sequential order (i.e., helix 1 is surrounded by helices 4 and 5 instead of helix 2). LacY is organized into two groups of six helices each (I to VI and VII to XII), and both groups are connected by a long loop between helices VI and VII. These groups of N- and C-terminal TMSs display two-fold symmetry. In addition, the N- and C-terminal halves present some weak similarities, suggesting gene duplication or fusion. Based on the known structure of LacY, we modeled the *T. cruzi* hexose transporter, which is a well characterized and representative member of the others analyzed in the present study. Interestingly, the model presented reliable adjusting parameters, with an e-value of 1.9×10^−27^ (Figure S3). Based on their structural similarity, we expected that evolutionary modules would correspond to neighboring TMSs, but this was not the case. This finding could be explained by the fact that, differently from what was predicted for human GLUT1, it was not expectable that trypanosomatid hexose transporters interact with other proteins: the only human GLUT1 known interactor (beside itself [43]) is GLUT1CBP [44], which is absent in trypanosomatids.

The N- terminus, TMS-1, L-1 and the C- terminus were generally the most divergent regions of GLUT proteins. In fact, the large extracellular loop between helices 1 and 2, present in all sequences, is one of the factors that diminished the quality of the LacY-based models. This result is not surprising because this feature, while common in the GLUT1 family, is absent in LacY [13]. When these regions were excluded, the resulting models showed that trypanosomatid transporters had a similar architecture to that of LacY (Figure S3). As discussed for Lac Y, two domains were present, constituted by helices 2 to 6 (corresponding to the N-terminal half of the molecule), and constituted by helices 7 to 12 (corresponding to the C-terminal half of the molecule). Based on the model, both halves seem to be linked by a long intracellular loop and surround a predicted cavity that opens to the cytoplasmic side of the membrane. It is also assumed that both the N- and C-termini are intracellular.

The differences in the role of transmembrane regions of most metabolite transporters, which are critical for their correct insertion into the membrane, as well as for their conformation and activity [7], led us to analyze if these TMSs should be more constrained during evolution than the loops which seem to be more prone to amino acid substitutions. Indeed, the only predictable evolutionary constraint for the loops would be related to the avoidance of highly immunogenic structures on the parasite's surface. The hexose transporters provide a very interesting and biologically relevant model to test the hypothesis that intramolecular evolutionary modules exist. Initially, we hypothesized that the TMSs would act as individual modules. However, the phylogenetic tree obtained with the TMSs showed a more complex pattern, including a positional clustering of consecutively ordered sequences (TMSs 1 and 2 or 11 and 12), strongly supporting the idea that these modules could originate via duplication. However, non-positional clustering (i.e., of TMSs that are not necessarily contiguous) was also observed. This finding could be explained by possible interactions between non-contiguous TMSs as reported for LacY.

The arrangement of the genes coding the trypanosomatids hexose transporters in their respective genomes was also analyzed. An interesting novelty is the fact that some trypanosomatids' transporters are expanded into multiple tandem duplications, and most of them are syntenic. This fact is contrasting with what is found in the ortologs from other organisms, like the human or mouse GLUT1, both as single-copy genes located in the chromosome 1 and 4, respectively, or *Drosophilla melanogaster* GLUT1, located in the chromosome 3 L, in all cases flanked by different pseudogenes. In synthesis, the genomic context is not conserved at all.

To summarize, as observed for the full-length sequences, phylogenetic analysis of the TMS, TMSJ and LJ sequences produced two major clusters that separated the sequences by genus. However, the phylogenetic trees constructed with the individual TMS sequences displayed the lowest mean distances, indicating that the TMSs are highly similar between proteins. This fact, together with the results obtained from the phylogenetic analysis, strongly supports the hypothesis that the loops are variable regions that are subject to the selective pressure of the immune system of the host. The TMSs, in contrast, are more constrained in terms of variation because they constitute the structural basis for the transport activity of the protein. The phylogenetic analysis of the pool of individual TMSs also showed their segregation in superclusters, some of which contain contiguous TMSs. This result indicates that the TMSs are positionally conserved and also suggests that the TMSs originated via intramolecular duplications. Nevertheless, the screening of the full-length sequences for internal repeats failed to detect consistently repeated sequences in the analyzed hexose transporters. The lack of data supporting duplications led us to conclude that the ancestral trypanosomatid already had a hexose transporter with multiple TMSs. However, we cannot rule out the occurrence of these kinds of events, which were reported as frequent in the original formation of the modules constituting these molecules.

## Supporting Information

Figure S1 **Evolutionary tree using the pool of individual L sequences.** A maximum likelihood method based on the JTT matrix-based model was used, with 500 bootstrap replicates. The tree is drawn to scale, with branch lengths measured in the number of substitutions per site.
(DOC)

Figure S2 **All 18 sequences were analyzed using the Multiple Em for Motif Elicitation (MEME) algorithm (** http://meme.nbcr.net/ **).** This analysis identified the motif QLTGINA (or the “GINA" motif), which was primarily present in TMS-6 and TMS-7. **A.** Sequence logo of the GINA motif. **B.** Conservation of the GINA motif in the analyzed sequences. **C.** Position of the GINA motif in the sequences.
(DOC)

Figure S3 **The** ***T. cruzi*** **hexose transporter sequence was modeled based on the known structure of the Major Facilitator Superfamily (MFS) member LacY from** ***E. coli*** **.** Wire models of both transporters (*T. cruzi:* blue; *E. coli* LacY: yellow) are shown, with the *T. cruzi* structure overlaid on the *E. coli* structure. Both molecules appear to be heart shaped when represented in a view parallel to the membrane. The red arrow indicates the direction of movement of the substrate (from the extracellular to the intracellular side).
(DOC)

Table S1 **The number of amino acid substitutions per site between sequences is shown.** Analyses were conducted using the JTT matrix-based model [21]. The analysis involved 216 amino acid sequences. All ambiguous positions were removed for each sequence pair. A total of 34 positions were included in the final dataset. Evolutionary analyses were conducted using MEGA5 [22].
(XLS)

Dataset S1 **Dataset of sequences in FASTA format.**
(RAR)
