# Correction: On the Evolution of Hexose Transporters in Kinetoplastid Potozoans

**DOI:** 10.1371/annotation/9773af53-a076-4946-a3f1-83914226c10d

**Published:** 2012-07-09

**Authors:** Claudio Alejandro Pereira, Ariel Mariano Silber

The word "Protozoans" is misspelled in the article title. The correct title is: On the Evolution of Hexose Transporters in Kinetoplastid Protozoans. The correct citation is: Pereira CA, Silber AM (2012) On the Evolution of Hexose Transporters in Kinetoplastid Protozoans. PLoS ONE 7(5): e36303. doi:10.1371/journal.pone.0036303

