# Supplementary material for: On the Evolution of Hexose Transporters in Kinetoplastid Potozoans
Source: PLoS One. 2012 May 2;7(5):e36303. doi: 10.1371/journal.pone.0036303 (PMC3342237; doi:10.1371/journal.pone.0036303)
Supplement: Figure S1 — Evolutionary tree using the pool of individual L sequences. A maximum likelihood method based on the JTT matrix-based model was used, with 500 bootstrap replicates. The tree is drawn to scale, with branch lengths measured in the number of substitutions per site. (DOC) [file pone.0036303.s001.doc]

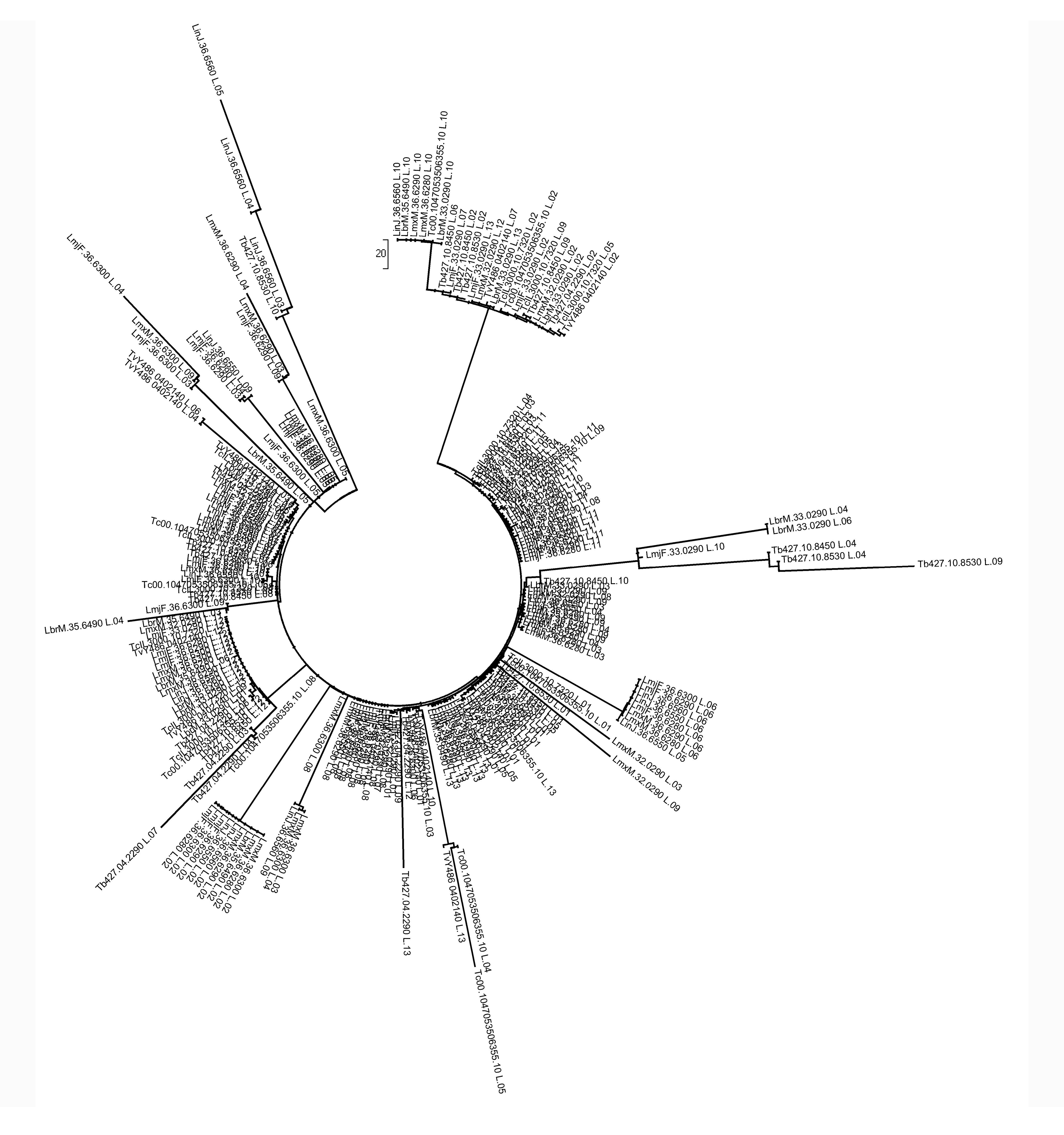


**Figure S1:** Evolutionary tree using the pool of individual L sequences. The Maximum Likelihood based on the JTT matrix-based model was used, with a bootstrap of 500 replicates. The tree with the highest log likelihood (-8883.9605) is shown. The tree is drawn to scale, with branch lengths measured in the number of substitutions per site.
