# Supplementary figures and images for: On the Evolution of Hexose Transporters in Kinetoplastid Potozoans
Source: PLoS One. 2012 May 2;7(5):e36303. doi: 10.1371/journal.pone.0036303 (PMC3342237; doi:10.1371/journal.pone.0036303)

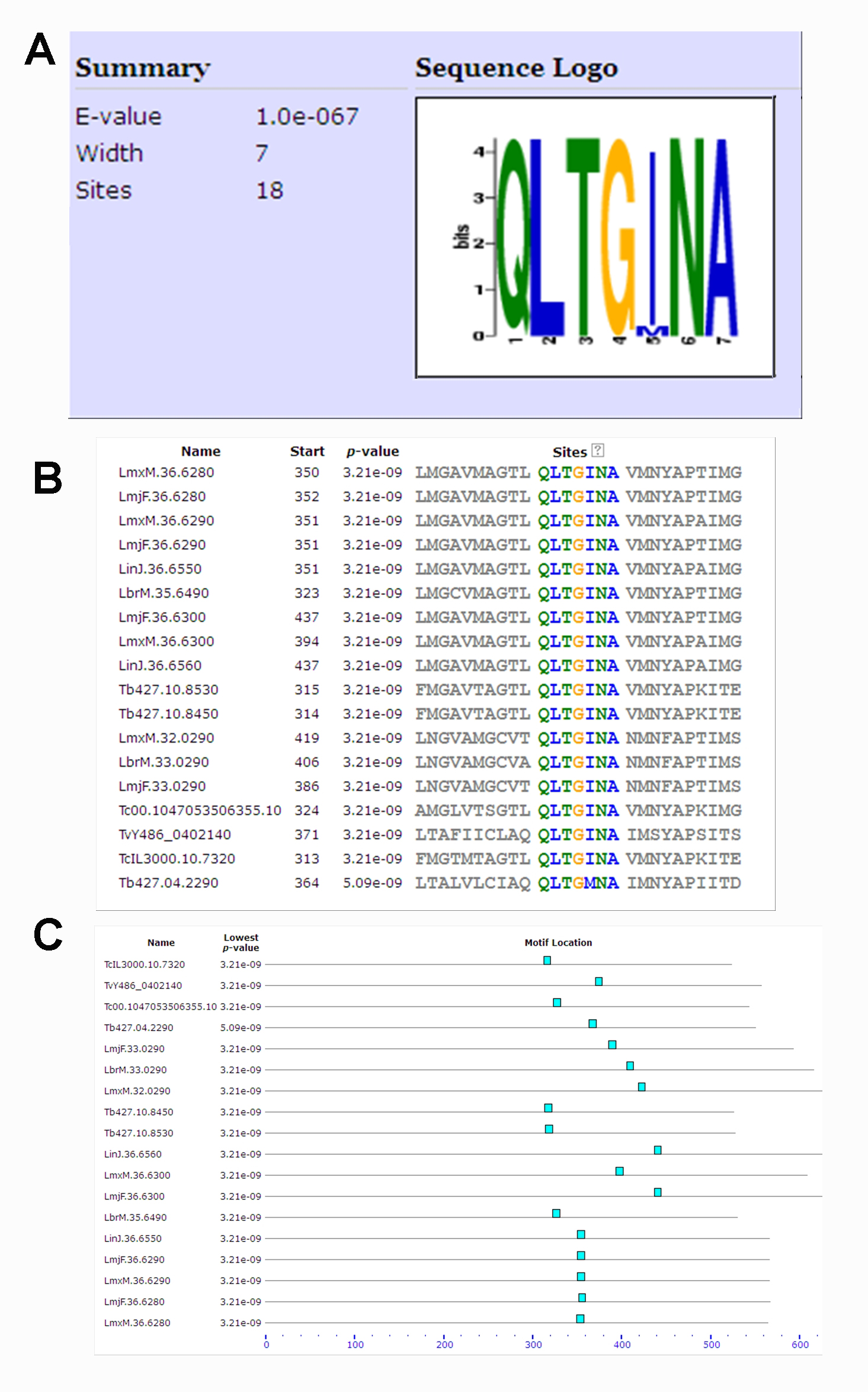

Supplement: Figure S2 — All 18 sequences were analyzed using the Multiple Em for Motif Elicitation (MEME) algorithm ( http://meme.nbcr.net/ ). This analysis identified the motif QLTGINA (or the “GINA" motif), which was primarily present in TMS-6 and TMS-7. A. Sequence logo of the GINA motif. B. Conservation of the GINA motif in the analyzed sequences. C. Position of the GINA motif in the sequences. (DOC) [file pone.0036303.s002.doc]
