# Supplementary material for: On the Evolution of Hexose Transporters in Kinetoplastid Potozoans
Source: PLoS One. 2012 May 2;7(5):e36303. doi: 10.1371/journal.pone.0036303 (PMC3342237; doi:10.1371/journal.pone.0036303)
Supplement: Figure S3 — The T. cruzi hexose transporter sequence was modeled based on the known structure of the Major Facilitator Superfamily (MFS) member LacY from E. coli . Wire models of both transporters (T. cruzi: blue; E. coli LacY: yellow) are shown, with the T. cruzi structure overlaid on the E. coli structure. Both molecules appear to be heart shaped when represented in a view parallel to the membrane. The red arrow indicates the direction of movement of the substrate (from the extracellular to the intracellular side). (DOC) [file pone.0036303.s003.doc]

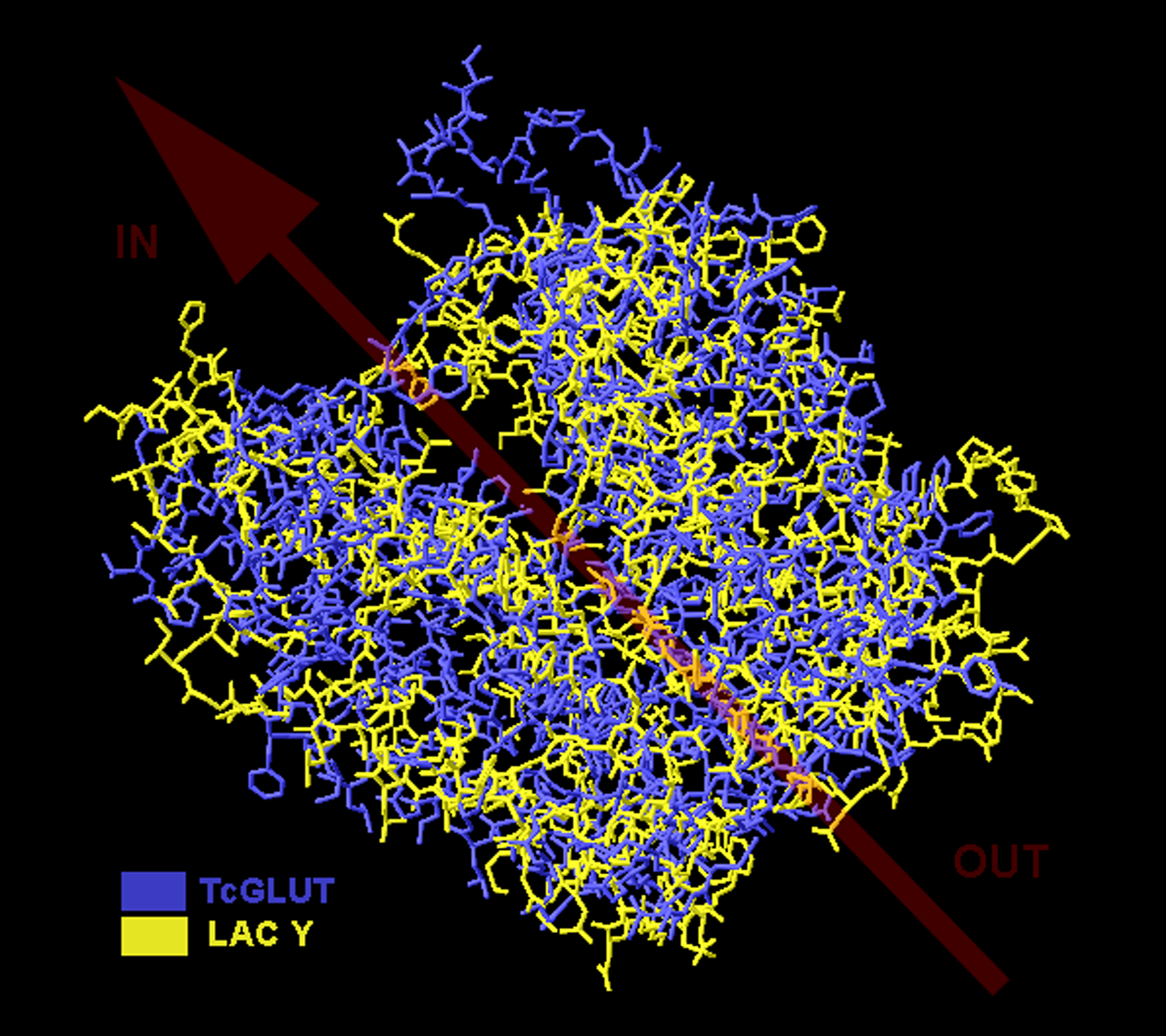


**Figure S3:** The *T. cruzi* hexose transporter sequence was modeled based on the known structure of the Major Facilitator Superfamily (MFS) member LacY from *E. coli*. Wire models of both transporters (*T. cruzi:* blue; *E. coli* LacY: yellow) are shown, with the *T. cruzi* structure overlaid on the *E. coli* structure. Both molecules appear to be heart shaped when represented in a view parallel to the membrane. The red arrow indicates the direction of movement of the substrate (from the extracellular to the intracellular side).
